# Reducing the time delay of oxygen transport to the neonate on continuous positive airway pressure support: A bench study

**DOI:** 10.3389/fped.2023.1141432

**Published:** 2023-04-19

**Authors:** Leos Tejkl, Petr Kudrna, Jakub Rafl, Thomas E. Bachman

**Affiliations:** Department of Biomedical Technology, Faculty of Biomedical Engineering, Czech Technical University in Prague, Kladno, Czech Republic

**Keywords:** neonatal oxygen transport, oxygen support, respiratory system, oxygen delivery, CPAP

## Abstract

**Background:**

Premature newborns often require oxygen support as part of their therapy. Systems for oxygen administration are developed to assure adequate oxygenation of newborns. Several factors were identified in the systems that contribute to the time delay between the change in the set inspiratory oxygen fraction and its actual delivery to tissues. In this study, we aimed to reduce the physical delay in oxygen delivery to newborns.

**Methods:**

We developed an O_2_ Flush System (O_2_-FS) that brings the source of oxygen as close to a patient as possible to make oxygen available for rapid delivery that compensates for the physical delay in the ventilator circuit. The O_2_-FS system is built around an electromechanical on/off valve. We validated the O_2_-FS concept in experiments with non-invasive Continuous Positive Airways Pressure (CPAP) ventilators.

**Results:**

The O_2_-FS accelerated oxygen delivery with all the tested systems and arrangements, typically by 5–15 s. We also observed that the application of supplemental oxygen increased the pressure in the ventilator circuit by 3–4 cmH_2_O which may mitigate the apneic pauses that are common in premature newborns.

**Conclusions:**

The O_2_-FS system may work as a universal accessory of the CPAP lung ventilator and shorten the distribution of oxygen to the patient during oxygen desaturation events, possibly eliminating or interrupting apneic pauses in neonates, for whom oxygen therapy is an essential treatment. In clinical practice, the O_2_-FS could help maintain normoxemic saturation values through adequate oxygen dosing in preterm neonates, thus reducing morbidity and mortality.

## 1. Introduction

The life of a preterm neonate often depends on respiratory support, comprised of supplemental ventilation and oxygen (1, 2). The former is needed as their lungs are not sufficiently developed and tend to collapse. The latter is because of the immaturity of the respiratory arterial interface and vascular system. The long-term goal of oxygen therapy is to maintain a normal value of partial pressure of oxygen in arterial blood (PaO_2_) in the range of 45–70 mmHg, which ensures aerobic metabolism and suitable conditions for proper organ development (1, 2). Premature newborns require higher amounts of oxygen in the inspiratory mixture to maintain adequate oxygenation of tissues and organs. Reduced concentration of oxygen in arterial blood (hypoxemia) is closely related to insufficient oxygen in the tissues (hypoxia) that causes slower organ development, increased pulmonary hypertension, and poses a risk of reopening of the arterial duct, resulting in mixing of arterial and venous blood.

The method of providing oxygen therapy depends on the origin of the hypoxemia and the patient's medical condition. The ventilation mixture, which must be optimally warmed and humidified, is administered into the airways either by inhalation or by insufflation (3). Inhalation is preferred in immature neonates who have preserved spontaneous respiratory activity as it reduces the risk of barotrauma. Oxygen is delivered either directly into the incubator or *via* the orofacial mask and nasal cannula. Insufflation is applied in neonates with insufficient spontaneous breathing using either mechanical lung ventilation or, if the neonate's medical condition permits, the non-invasive modes such as CPAP (Continuous Positive Airways Pressure) and HFHHNC (High Flow High Humidity Nasal Cannula). The physiological effect of CPAP contributes to an increase in the partial pressure of oxygen in the arterial blood (4). Oxygen therapy utilizes blenders that allow the oxygen fraction of the inspiratory mixture (FiO_2_) to be set between 21% and 100% as part of the ventilatory support. The flow rate of the ventilation mixture delivered to the patient can range from 0.01 L/min to 15 L/min.

In neonatal oxygen therapy, efforts are made to avoid hyperoxaemia (3, 5). Increased oxygen in the tissues (hyperoxia) due to increased concentration of oxygen in arterial blood causes disproportionate amounts of oxygen radicals that increase the risk of oxidative stress (1). Oxygen negatively affects the lungs and can cause chronic lung disease in the form of bronchopulmonary dysplasia (BPD). It also impairs the development of the nervous system, especially in the retina, by forming retinopathy of prematurity (ROP). Therefore, proper adjustment of oxygenation within a narrow range of values is very important in neonates (1, 3, 6). Monitoring of oxygenation is commonly performed using pulse oximeters, which continuously and non-invasively measure the peripheral blood oxygen saturation (SpO_2_) (7).

The adjustment of FiO_2_ is classically performed manually by the attending staff using mechanical and electromechanical blenders. The trend is toward automatic control of oxygenation in ventilators using a feedback control algorithm (8). These exclusively use the electromechanical blender, which is only supervised by the attending staff, but the actual adjustment of the inhaled oxygen fraction is performed automatically by the control algorithm based on the measured SpO_2_ values. Many studies confirm improved compliance in the normoxemic range and time savings for nursing staff (8, 9).

**Figure 1:**
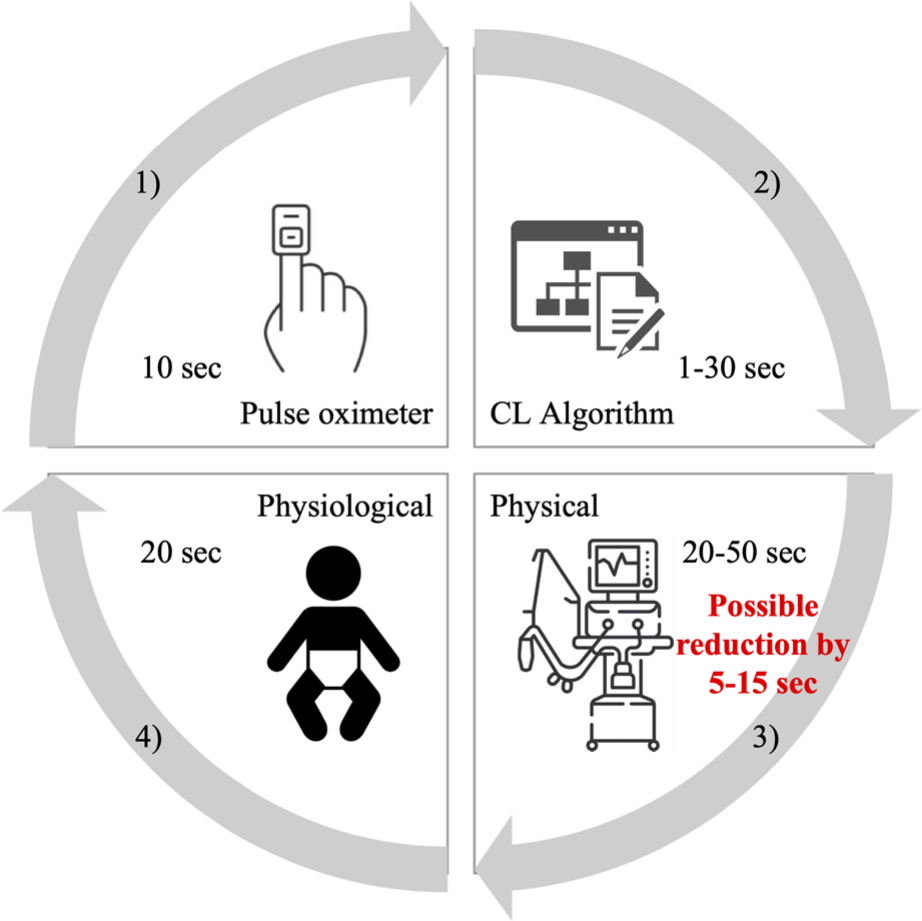
Schematic of the delays contained in automatic feedback control systems of oxygenation. These are delays by (1) pulse oximeter averaging, (2) CL algorithm evaluation, (3) physical delays, and (4) physiological processes in the body. The red numbers indicate the expected possibility of reducing the delay in oxygen distribution to the patient caused by technical deficiencies.

Fathabadi (10) and Krone (11) characterized the change in SpO_2_ following a change in FiO_2_ by three parameters—the delay, time constant, and gain (10). The median observed time delay in (10) was 22 s and the median of three times the time constant was 39 s. According to published interquartile ranges, the total delay is between 15 and 145 s. These values include technical and physiological delays. In feedback control systems, several factors contribute to the time delay as shown in Figure 1. First, when measuring SpO_2_ with a pulse oximeter the signal is averaged. For example, in the Rainbow system with the Radical 7 pulse oximeter (Masimo Corp., USA) the signal averaging can be set to 2–4, 4–6, 8, 10, 12, 14, or 16 s with a preset value of 8 s (12). Second, the control algorithm itself, depending on its type, can have a time delay of over a minute. A study does suggest that faster adjustments, whether manual or automated, are better (13). Automatic systems evaluate SpO_2_ in a certain time interval. Systems with fast changes may exhibit instability, but slow and stable systems may not respond adequately. CLAC (Close-Loop Automatic Oxygen Control, Heinen + Löwenstein Lebenserhaltende Medizintechnik, Hamburg, Germany) has two versions, with the fast version performing setup every 30 s and the slower configuration every 180 s (14). The PRICO (Vyaire Medical, Inc., Mettawa, United States) system changes the oxygen fraction setting every 30 s, while other systems consider adjustments every second (15). Third, a part of the time delay is related to the properties of the blender and the ventilator circuit. This physical delay has been measured to be between 20 and 40 s (16, 17). Finally, fourth, the physiological delay in the distribution of oxygen depends on many physiological factors, including the active alveolar surface area, diffusion across the alveolar-capillary membrane, and the state of the cardiovascular system (e.g., blood volume, Hb concentration, HbF/HbA ratio, cardiac shunts, peripheral circulation, and other factors affecting oxygen flow). Delays are higher tens of seconds (1, 10, 18).

To move towards an optimum, a fast and efficient feedback control loop oxygen adjustment system, we focused on areas of improvement. One area is reducing unnecessary delays in the control loop. An engineering solution that would reduce the physical delay caused by technical shortcomings of ventilatory support would offer an opportunity to accelerate the oxygen distribution to the patient and could play a significant clinical role in future systems for neonatal oxygenation.

The aim of this work was to reduce the physical delay in the distribution of oxygen to the neonates during ventilatory support. In this article, we present the conceptual design, hardware and software implementation, and validation of a universal prototype device that reduces the time delay in oxygen distribution to the patient following a change in the inspiratory oxygen.

## 2. Concept of O_2_ flush system

We proposed a system to reduce the physical delay in oxygen distribution to the neonate after a change in FiO_2_, further referred to as the O_2_ Flush System (O_2_-FS). In principle, the system delivers an additional burst of oxygen (at a constant volume flow rate of 2 Lpm) into a ventilator circuit as close to the patient as possible while minimizing interference with the ongoing ventilatory support. This is achieved by a series of pneumatic elements that bypass the standard ventilator circuit and feed oxygen directly to the patient during CPAP. The combination of pneumatic elements also assures the pressure in the system is limited to a value that does not harm the patient and eliminates pressure fluctuations when opening and closing an electronically controlled valve. The O_2_-FS consists of pneumatic hardware, electronic hardware, and control software.

### 2.1. Hardware design and implementation

**Figure 2:**
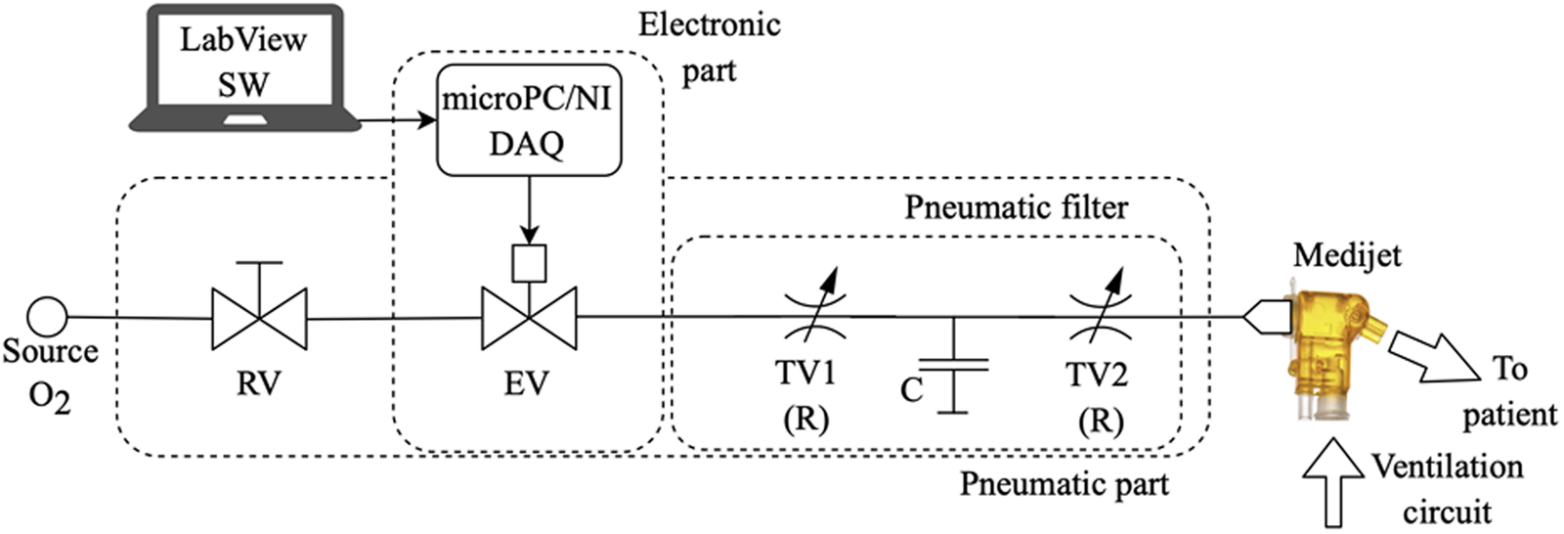
The schematic arrangement of the O_2_-FS in configuration with the Medijet CPAP interface. The same principle applies in the case of the Infant Flow or Infant Flow Low Pressure interfaces. Symbols: RV, reducing valve; EV, electromagnetic controlled valve; TV1 and TV2, throttle valves; C, pneumatic compliance.

The schematic arrangement of the O_2_-FS is presented in Figure 2. The system is connected to the oxygen delivery system *via* a reducing valve that reduces the oxygen pressure to 1 bar. After the pressure-reducing valve, there is an electromechanical on/off valve that controls the flow of oxygen. In our experimental device, we used the MHJ9-QS-4-MF valve with an external control element MHJ9-KMH-2,5-MF (Festo AG & Co, Esslingen am Neckar, Deutschland). A pneumatic filter follows, which consists of proportional throttle valves and pneumatic compliance, to smooth out pressure shocks that arise by opening/closing the valve. The compliance was implemented as a 5-cm elastomeric tube. The electronics contain a control module for the electromechanically controlled valve, a microcomputer, a power supply, and a control unit DAQ USB-6002 system (National Instruments, Austin, USA). The system has its battery backup power supply in case of disconnection from the electric network.

**Figure 3:**
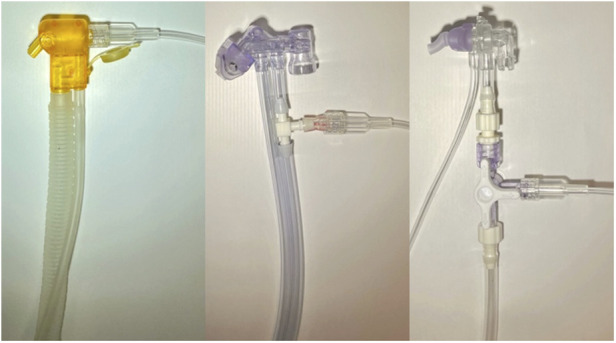
Connection of the O_2_-FS to CPAP interfaces. From left: Medijet, Infant Flow, and Infant Flow Low Pressure.

The connection of the O_2_-FS to the interface (also called the applicator by some manufacturers) is specific to the CPAP device as documented in Figure 3. For Medijet (Medin Medical Innovations GmbH, Olching, Germany), a Luer lock tube was attached to the nebulizer part of the interface. For Infant Flow or Infant Flow Low Pressure (Vyaire Medical, Inc., Chicago, United States), the original path of the ventilator circuit was interrupted by a three-way connector. The length of the ventilator circuit including the interface was 170 cm for Medijet and 180 cm for Infant Flow and Infant Flow Low Pressure. The length of the tubing between the interface of the nCPAP ventilator and the hardware of O_2_-FS was 80 cm with a volume of 1 ml.

### 2.2. Control software

**Figure 4:**
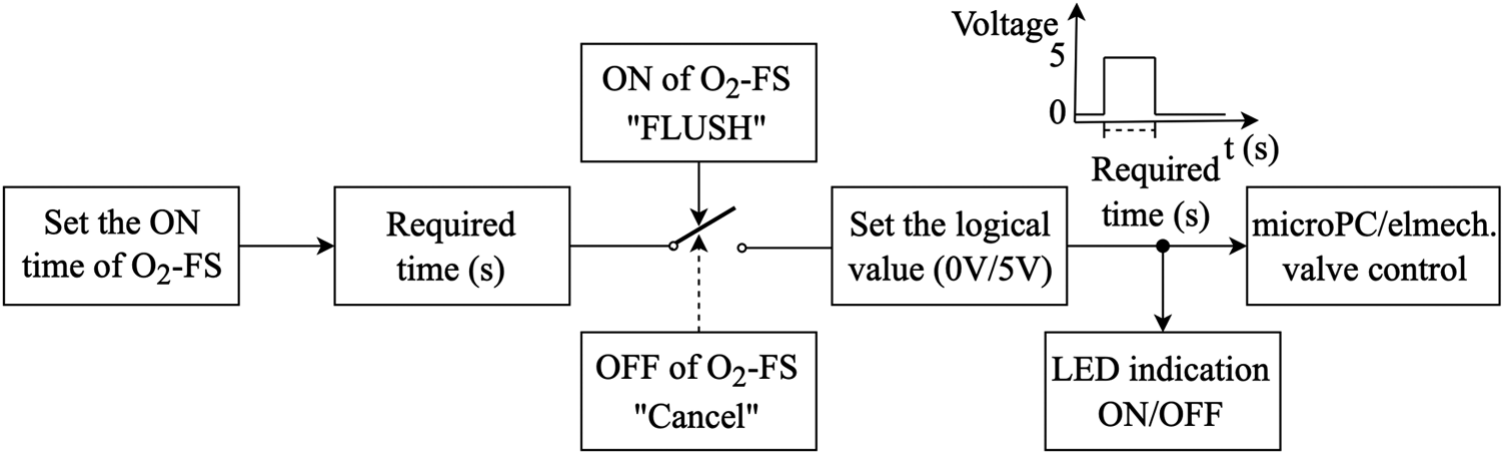
The schematic arrangement of the O_2_-FS operation.

The O_2_-FS control system was designed to allow both the preset duration of oxygen delivery and direct manual oxygen dosing. The operation of the control system is shown in the block diagram in Figure 4. Oxygen supply time (ON time) permits setting the duration of the oxygen delivery from 1 to 10 s. The oxygen supply is then manually initiated (FLUSH) but can be terminated anytime during the oxygen delivery period (Cancel). The system visually indicates if oxygen is being delivered.

## 3. System validation

We experimentally investigated how the O_2_-FS changed the oxygen distribution to the patient over time after FiO_2_ was changed and how the operation of the O_2_-FS affected pressure in the CPAP ventilator circuit (pCPAP). The experiment was conducted in laboratories of the Faculty of Biomedical Engineering in Kladno under standard laboratory conditions. The protocol of the experimental procedure validation was derived from our previous study that described the time delay in the distribution of oxygen in a ventilator circuit (16).

### 3.1. Experimental setup and protocol

We tested the O_2_-FS with the CPAP ventilators commonly used in neonatal ICUs in Europe. These were Fabian Therapy Evolution (Vyaire Medical, Inc., Mettawa, United States), Infant Flow SiPAP (Vyaire Medical, Inc., Mettawa, United States), and MedinCNO (Medin Medical Innovations GmbH, Olching, Germany) as summarized in Table 1. Where it was possible, we tested several combinations of a ventilator and a suitable CPAP interface. Before each measurement, a functional test of a combination of a ventilator and an interface was performed according to the manufacturer's instructions.

**Table 1:** CPAP ventilators and suitable interfaces used for validation of the O_2_-FS. The accuracy of ventilators is presented as reported by manufacturers.

CPAP ventilator	Type of blender	Accuracy (%)	Set parameter	Interfaces
Fabian	Elmech	3	Pressure pCPAP	IF, IFLP, MJ
SiPAP	Manual	3	Volume flow rate	IF, IFLP
MedinCNO	Elmech	3	Volume flow rate	MJ

IF, Infant Flow; IFLP, Infant Flow Infant Flow Low Pressure; MJ, Medijet; Elmech, Electromechanical.

**Figure 5:**
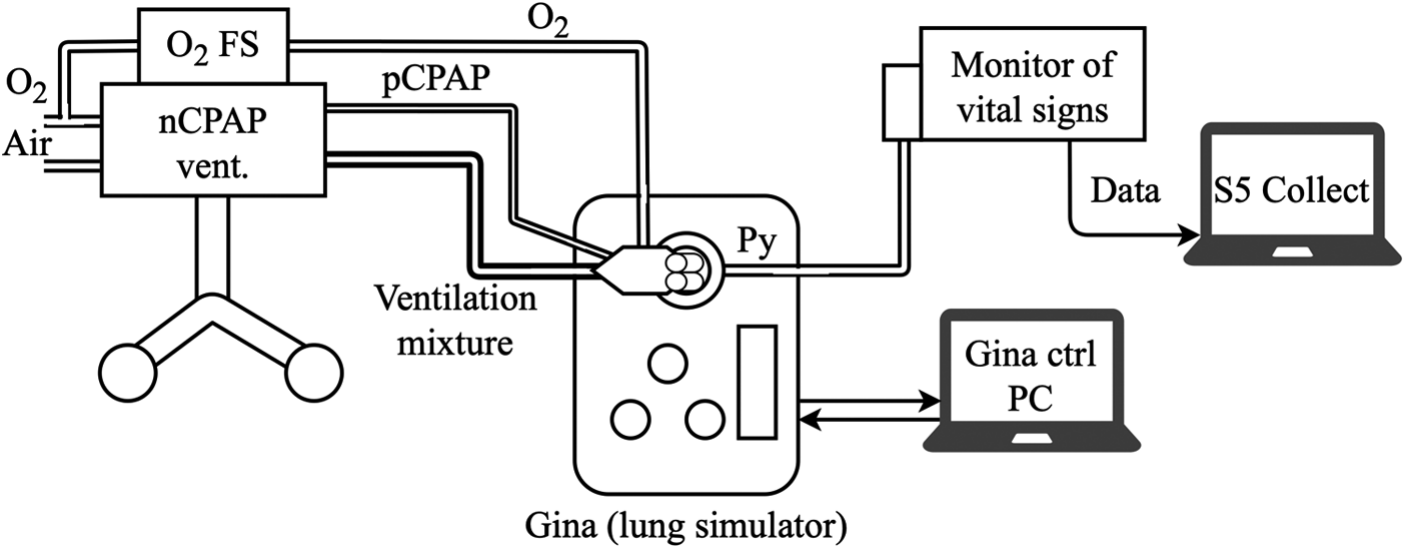
Experimental setup with a CPAP ventilator, O_2_-FS, nasal system interface, vital signs monitor, and active lung model for experimental validation of the O_2_-FS concept.

The experiment setup is presented in Figure 5. A CPAP ventilator, combined with an interface, was connected to sources of oxygen and compressed air and connected with the neonatal lung simulator. The O_2_-FS was attached to the ventilator circuit as described above. Original accessories associated with a specific CPAP ventilator were used wherever possible, but we had to use a custom-made fixture that allowed connection of the interface with nostrils to the neonatal lung simulator that has an inlet adapted to the endotracheal tube.

The neonatal active lung simulator Gina (Dr. Schaller Medizintechnik, Dresden, Germany) was used as a physical model of the preterm infant lung. The settings of the lung simulator parameters were the same for all measurements: apnea (no spontaneous breathing), Cint = 0.3 ml/hPa, lung volume = 10 ml, RETT = 5 (non-invasive ventilation), airway resistance Raw = Ra4, Leak off, Compliance = Cint, and the pressure measurement point Py at the Y junction. No humidifier was used or incorporated during the experiment. The nostril connectors for Infant Flow and Infant Flow Low Pressure were selected in size L. In the case of the SiPAP ventilator, which has a mechanical blender without a precisely marked scale, the dial positions corresponding to the desired oxygen fraction were identified and marked prior to measurement.

The oxygen fraction (mFiO_2_) was measured at the pressure measurement point Py (Figure 5) by the E-COVX respiratory module of the Carescape B650 patient monitor (GE Healthcare, Helsinki, Finland). The total response time delay of the respiratory module was 2.9 s and consisted of a sampling delay of 2.5 s and a rise delay of 0.4 s, as described in the user manual. Data from the patient monitor were collected using Datex-Ohmeda S/5 Collect software (GE Healthcare, Helsinki, Finland) and analyzed in Matlab R2021b (Mathworks, Natick, USA) with a maximum possible sampling rate of 1 Hz.

The CPAP ventilator was set for the required value of the CPAP pressure (Fabian) or volume flow rate (SiPAP and MedinCNO) and the set oxygen fraction (sFiO_2_). Initially, sFiO_2_ was set at 21% and then increased to 31% (set fraction change: *Δ*sFiO₂ =  + 10%). The moment the sFiO_2_ was increased, the O_2_-FS was switched on for 2 s. After mFiO_2_ stabilized at a new level, sFiO_2_ was set back to 21% and the measurement was repeated with the O_2_-FS switched on for 4 s. Both 2 s and 4 s measurements were then repeated for an sFiO_2_ increase from 21% to 51% (*Δ*sFiO_2_ =  + 30%). The moment of an sFiO_2_ increase and the activation of the O_2_-FS was deemed as time zero for the subsequent data analysis. Measurements were carried out for pCPAP levels from 2 to 12 cmH_2_O in steps of 2 cmH_2_O with the Fabian CPAP ventilator, or for gas flow rates corresponding to the pCPAP in the same steps with the volume-control CPAP ventilators. At the same time, pCPAP was measured in two ways before and after the activation of the O_2_-FS. First, pCPAP was recorded by the lung simulator and second, we manually took readings from the ventilator display.

### 3.2. Data processing and analysis

**Figure 6:**
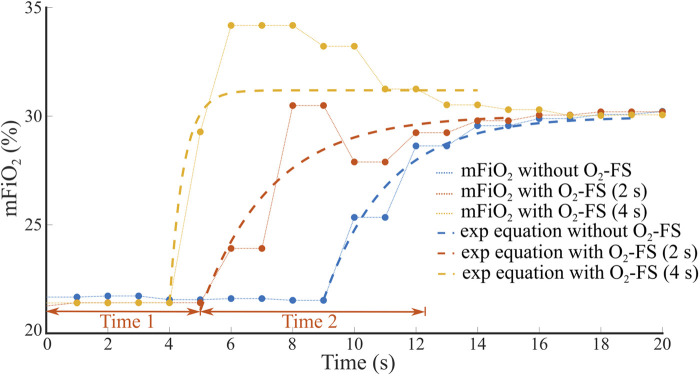
An example of the time delay of mFiO_2_ increase after sFiO_2_ change from 21% to 31%. Total time delay consists of the baseline delay (Time 1) and the duration of mFiO_2_ rise (Time 2) that was approximated by an exponential function. The set flow rate on the CPAP ventilator was 8 L/min.

The total time delay of an increase of mFiO_2_ was divided into two parts, Time 1 and Time 2, as shown in an example in Figure 6. Time 1 represents the baseline delay, where mFiO_2_ does not change, from time zero to the point where mFiO_2_ started to rise. The measured Time 1 was reduced by a 2.5-s sampling delay of the E-COVX module. Time 2 characterizes the duration of mFiO_2_ rise. In this part, the mFiO_2_ signal was modeled as an exponential function:

$$y(t)=a\left(1-e^{-\frac{t}{\tau }}\right)+b$$

where *t* represents time (s). The values of the time constant *τ* and the parameters *a* and *b* were found using the curve fitting algorithm (Curve Fitting Toolbox, Matlab, MathWorks, Natick, USA) to provide the best approximation of mFiO_2_ in the sense of the least square's method. Time 2 was estimated as 3*τ* and reduced by the 0.4-s rise delay of the E-COVX module.

The resulting total time delay was calculated as the sum of Time 1 and Time 2 and evaluated for each combination of a CPAP ventilator and interface, *Δ*sFiO_2_, and gas flow rate. For measurements with O_2_-FS activated, we averaged the total delays of the two configurations of the O_2_-FS (2 s and 4 s). The mean difference in the total delay with and without the O_2_-FS activated was evaluated by a paired t-test after the normality of the data was tested using the Shapiro-Wilk test; *p* value < 0.05 was considered statistically significant. The data were analyzed in Matlab R2021b (Mathworks, Natick, USA).

## 4. Results

**Figure 7:**
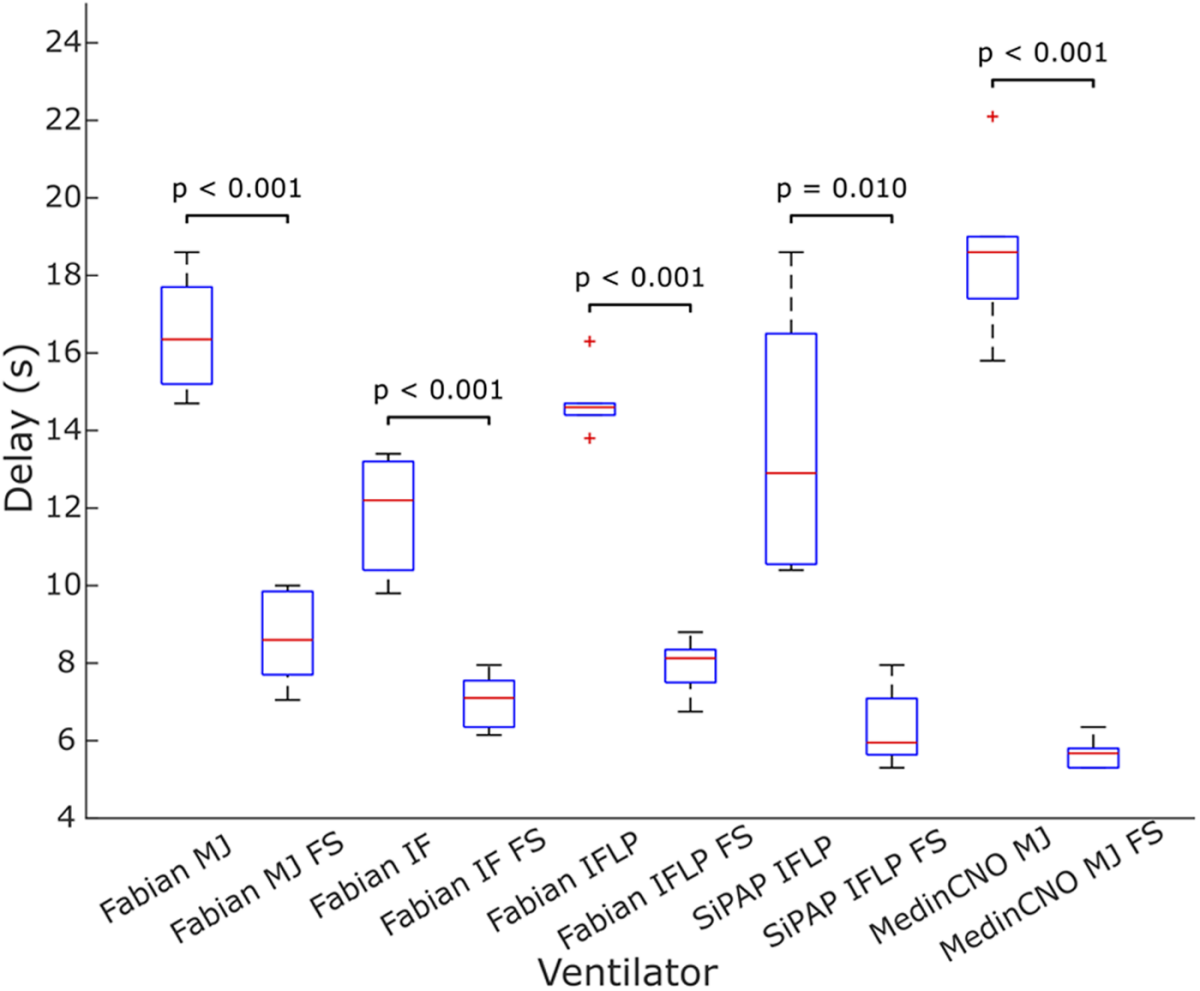
Delay in oxygen delivery upon a 10% increase in FiO_2_ without the O_2_-FS and with the O_2_-FS. Fabian MJ, Fabian with Medijet interface, Fabian IF, Fabian with Infant Flow interface, Fabian IFLP, Fabian with Infant Flow Low Pressure; SiPAP IFLP, Vyaire SiPAP with Infant Flow Low Pressure interface; Medin MJ, Medin MedinCNO with Medijet interface. FS marks a configuration with the O_2_-FS. Each box plot displays the following information: median (red line), lower and upper quartiles (blue rectangles), outliers (red crosses), and minimum and maximum values that are not outliers (black lines).

**Figure 8:**
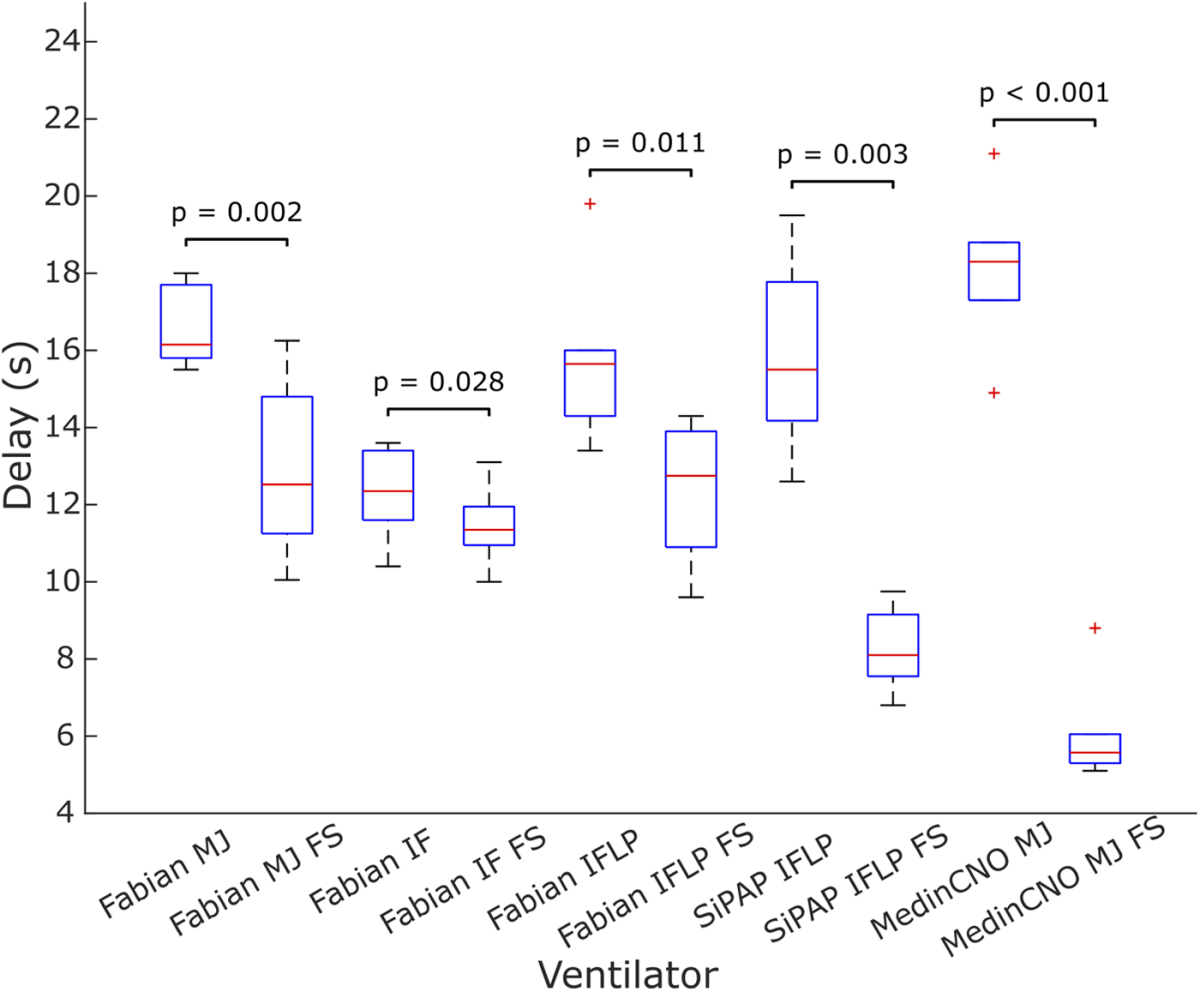
Delay in oxygen delivery upon a 30% increase in FiO_2_ without the O_2_-FS and with the O_2_-FS. Fabian MJ, Fabian with Medijet interface; Fabian IF, Fabian with Infant Flow interface; Fabian IFLP, Fabian with Infant Flow Low Pressure; SiPAP IFLP, Vyaire SiPAP with Infant Flow Low Pressure interface; Medin MJ, Medin MedinCNO with Medijet interface. FS marks a configuration with the O_2_-FS. Each box plot displays the following information: median (red line), lower and upper quartiles (blue rectangles), outliers (red crosses), and minimum and maximum values that are not outliers (black lines).

We experimentally evaluated the effect of the O_2_-FS on oxygen delivery upon increasing FiO_2_ in three different neonatal CPAP ventilators. Five combinations of a ventilator and an interface were tested. In all the configurations the O_2_-FS significantly reduced the delay of increased oxygen delivery when compared to a standard configuration of the ventilators as shown in Figures 7, 8. The average reduction in the time delay of oxygen delivery to the patient across the ventilators and interfaces used was 8.0 s (range 3.7–16.3 s) when FiO_2_ was increased by 10% and 5.4 s (range 1.3–15.8 s) when FiO_2_ was increased by 30%. The greatest difference was observed with the MedinCNO ventilator, where the delay in oxygen distribution to a patient was up to 20 s in a standard configuration but was reduced to 5 s when using the O_2_-FS. A smaller difference was observed when using the Fabian ventilator with three different interfaces, where the electromechanical blender is designed with two fast proportional valves. For the Infant Flow Low Pressure configuration of the Fabian ventilator, the delay without the O_2_-FS was smaller than in other configurations of the same ventilator due to the higher gas flow rate of the ventilation mixture was needed to maintain the same CPAP pressure for this configuration.

The CPAP pressure increased when supplemental oxygen was applied *via* the O_2_-FS, as summarized in Table 2. The pCPAP was elevated during the entire period when the O_2_-FS was operating. The median pCPAP increase value for all measurements was 3.9 cmH_2_O with an interquartile range of 3.0 cmH_2_O to 4.9 cmH_2_O.

**Table 2:** Comparison of pCPAP values measured in Fabian, SiPAP and Medin CPAP ventilator (with different interfaces) without the O_2_-FS (OFF) and with the O_2_-FS (ON).

pCPAP (cmH_2_O)															
Set CPAP	Fabian									SiPAP			Medin		
	MJ			IF			IFLP			IFLP			MJ		
	OFF	ON	Δ*p*	OFF	ON	Δ*p*	OFF	ON	Δ*p*	OFF	ON	Δ*p*	OFF	ON	Δ*p*
2	1.7	4	2.3	1.8	4.8	3.0	1.9	5.7	3.8	1.8	4.8	3.0	1.5	3.9	2.4
4	3.8	7	3.2	3.9	7.8	3.9	4.0	7.8	3.8	3.9	7.6	3.7	3.3	6.2	2.9
6	5.9	9	3.1	6.0	9.9	3.9	5.9	11.0	5.1	5.9	8.6	2.7	5.6	9.6	4.0
8	7.9	12	4.1	8.2	12.4	4.2	7.9	13.0	5.1	8.0	10.8	2.8	7.4	13.2	5.8
10	9.9	**15**	5.1	10.3	**15**.**0**	4.7	9.8	14.5	4.7	10.0	**11**.**7**	1.7	9.6	**15**.**4**	5.8
12	11.9	**17**	5.1	12.5	**16**.**8**	4.3	12.0	**15**.**2**	3.2	n/a	n/a	n/a	11.7	**18**.**4**	6.7

MJ, Medijet; IF, Infant Flow; IFLP, Infant Flow Low Pressure; Δ*p* = pCPAP(O_2_-FS ON) – pCPAP(O_2_-FS OFF), delta pressure in the CPAP ventilator circuit. The bold values mark the situation when pCPAP triggered the safety range of a ventilator.

## 5. Discussion

This study demonstrates that there is a feasible technical solution to the problem of the delay of oxygen distribution to a neonate after a change in a set FiO_2_. Previous studies found a significant delay between the change in the set FiO_2_ and the actual oxygen delivery to neonates (16, 17). However, we demonstrated that a system with a controlled electromechanical valve that delivers extra oxygen directly to the point where a non-invasive ventilatory support interface connects to a ventilator circuit reduces the delay of oxygen distribution to the neonate significantly. This should be equally applicable to invasive ventilation. Reducing the delay of oxygen distribution is also relevant in both manual and automatic oxygenation feedback control systems. The O_2_-FS could aid conventional ventilators with manual control where automatic control is not available. When used with a manually controlled ventilator, it will help keep the patient in the normoxemic range with successive small doses of oxygen until the ventilator operator arrives and changes the oxygen fraction. Many studies describe the faster response of automatic systems compared to manual settings (8). However, the O_2_-FS may reduce delays for automated systems to work even more efficiently. Our module could help to better maintain normoxia in preterm newborns.

The available feedback control systems adjust FiO_2_ at 1–180 s intervals and the total time for oxygen distribution delivery is estimated about 50–110 s (as illustrated in Figure 1). Accelerating oxygen delivery by 5–15 s, corresponding to approximately 5%–30% reduction in the total oxygen distribution time, may help even automated systems to better manage the oxygenation of preterm infants as some desaturation episodes can be shortened and mitigated, but, on the other hand, may not be of clinical significance. The clinical relevance of using O_2_-FS varies according to the specific ventilator and its electromechanical blender. It would have a greater effect for the medinCNO ventilator with an electronically driven blender than for Fabian Therapy Evolution with its blender controlled by two proportional valves.

We validated our concept using the full spectrum of available non-invasive (CPAP) ventilators with recommended interfaces across the full spectrum of adjustable pressures (flow rates), including the low nCPAP which admittedly has little clinical relevance. The measured delays without O_2_-FS matched those measured in our earlier study (16). It could be concluded that the connection of the system in the off state does not affect the normal function of the CPAP ventilatory support. Moreover, from a technical point of view, the O_2_-FS is a universal module that can be also connected to invasive ventilatory support when the patient is intubated. In any case, the system should be placed near the CPAP ventilator (as we did) or on a holder near the patient, as any added dead space compromises the effectiveness. Ultimately it would be integrated into the ventilator. A similar technical solution was described only by Rodriguez et al., who delivered additional oxygen to the Y-coupling of the ventilator circuit during adult invasive ventilation using a portable oxygen concentrator (19). They investigated the most efficient delivery of oxygen to patients in situations when compressed medical oxygen is not available. Injection of oxygen into the ventilation circuit upstream of the Y-coupling using pulse dosing with tidal volume correction was evaluated as the most effective method. However, this study was not concerned with the rate of oxygen delivery, but only with achieving the highest possible oxygen fraction using an oxygen concentrator.

We observed a pressure increase in the ventilator circuit caused by a higher flow rate of the ventilation mixture when the O_2_-FS was switched on. The pressure increase during the application of supplemental oxygen was in the range 3–4 cmH_2_O. These can potentially interrupt apneic pauses, which are a common cause of desaturations. Tactile stimulation of newborns by external pressure is known to be very effective in interrupting apneic pauses and restoring the breathing activity of preterm infants (20, 21). There are ventilation systems that take advantage of this effect such as “ApneaCPAP mode” used in MedinCNO ventilators which automatically increases the flow rate during apnea and stimulates breathing by so-called APAP (Automatic Positive Airways Pressure) (22). In our experiments, the pressure increases in the CPAP ventilation system depended not only on the gas flow rate/pressure set but also on the pressure-flow characteristics of interfaces, in which different CPAP pressures are achieved for a given gas flow rate, as described in (23).

During the measurements, we also observed a difference in effectiveness in improving the oxygen fraction depending on the magnitude of the set gas flow rate and pressure in the CPAP system. At lower gas flow rates, the effect of O_2_-FS was more marked because a relatively higher flow rate increases in the interface compartment due to O_2_-FS action. Without O_2_-FS, it took a long time for the ventilation mixture of the desired fraction to reach the patient.

### 5.1. Limitations

The system could not be validated on preterm neonates for ethical and safety reasons. A neonatal pulmonary simulator was used with the parameters of a preterm. In our simulations we achieved the desired reduction in the delay of oxygen distribution to the patient and validated the new technical solution in a proof-of-concept sense; nevertheless, all simulations have limited fidelity and complexity and our study did not (and did not aim to) show how the proposed system interacts with a real patient.

We did not use a humidifier during our experiments as the lung simulator cannot work with a gas mixture with saturated water vapor. The humidifier would have increased the volume of the ventilator circuit and thus increased the time delay of O_2_ change. It follows that our results presented for the configuration without the O_2_-FS were reached in the configuration that provides the fastest response of the CPAP ventilators to FiO_2_ change and greater differences between delays without and with the use of the O_2_ FS could be expected because humidifier would not interfere with the O₂-FS.

For testing purposes, the throttle valves were tuned for optimal operation at CPAP pressures of 6–8 cm H_2_O, which are typically used in clinical practice. Thus, switching on the O_2_-FS at the highest CPAP pressures of 10–12 cmH_2_O led on some occasions to the pressure safety limit being exceeded and the ventilatory support was automatically interrupted for a short time. In this case, the effect of the O_2_-FS disappeared before the ventilator resumed its function and reestablished the positive overpressure. However, we aimed to design a proof-of-concept system to reduce the delay of oxygen distribution to the patient, not to optimize the system for a specific ventilator. To eliminate this deficiency, the use of electronic throttling valves and their software settings for the desired pressures (flow rates) with respect to a specific ventilator type would be required.

The measurement at low flow rates/pressures resulted in an overshoot of the desired oxygen fraction value as illustrated in Figure 6. The set FiO_2_ change from 10% to 31% percent at pressure 2 cmH_2_O resulted in an actual oxygen concentration measurement of up to 50% for a very short period of 2–3 s and then stabilized at the set FiO_2_ value. The volume of oxygen supplied by the O_2_-FS system was typically between 60 and 120 ml, which accounts for only 20% of the total volume delivered for 2 to 4 s. We speculate that this overshoot would not have any adverse clinical effect due to short time and a very small amount of excess oxygen relative to the total gas supply to the patient. The overshoot may be better described by the second-order response function rather than the exponential. We preferred the exponential fit due to it was used in previous studies (10, 16, 17) and for this study we considered the overshoot relatively unimportant compared to the rise time described by the time constant.

### 5.2. Further development

Further developments of the presented concept can be considered in several directions. Firstly, the system can be optimized for a specific ventilator and interface to avoid the shortcomings related to synchronization and overshooting of the desired oxygen fraction. Practically, the O_2_-FS would benefit from the data communication with the ventilator to obtain information about the current flow, pressure, and FiO_2_ setting or be integrated into an automated FiO_2_ control system. Electronically controlled throttling valves could adjust the volume flow rate of oxygen in the O_2_-FS according to the actual flow rate of the ventilation mixture in the standard ventilator circuit and the oxygen flow rate delivered by the O_2_-FS could better adapt to the set CPAP pressures and flow rates. As a result, the required FiO_2_ will not be exceeded and an inadequate increase in interface pressure will not occur.

Alternatively, another option is to make the O2-FS function fully automatic. The O_2_-FS could be interfaced with a pulse oximeter. Based on the SpO_2_ reading, the system would assess whether an increase in FiO_2_ is required and act if appropriate, dosing small amounts of oxygen during initial desaturation and thus preventing an episode of deep desaturation, and perhaps end an apneic pause. This solution could be of great benefit in developing countries and in places where there are insufficient resources to purchase fully automatic oxygenation management systems and excessive and inadequate oxygenation management causes excessive morbidity and mortality. In the case of ventilators with automatic feedback control, this might help to better overcome the gap to the next controller intervention which may be up to 30 s and the additional delay in their ventilator circuit.

## 6. Conclusion

Our concept of the O_2_-FS addresses the general problem of the delay of oxygen distribution to neonates. The O_2_-FS is intended as a versatile automatic accessory to non-invasive ventilatory support, not available on the current market. Such a module can augment the function of a conventional ventilator with either manual or automatic control, accelerating the change of oxygen fraction at the airways to the desired level. In this proof-of-concept bench study, the prototype of the O_2_-FS we designed and implemented reduced the delay of oxygen distribution to the patient and at the same time added the possibility of automatic elimination or interruption of apneic pauses due to short-time pressure changes. However, it would be essential to validate these findings during clinical testing with the next version of the system.

## Data Availability

The raw data supporting the conclusions of this article will be made available by the authors, without undue reservation.

## Author's note

The technical essence of the solution is protected as a utility model PUV 2020-38066 by the Industrial Property Office of the Czech Republic.
